
JOURNAL INFORMATION
==============================
NLM Title Abbreviation: ScientificWorldJournal
Journal ID: ScientificWorldJournal
NLM Journal ID: 101131163
Journal ID: TSWJ

The Scientific World Journal

ISSN: 2356-6140
EISSN: 1537-744X
Publisher: Wiley

ARTICLE INFORMATION
==============================
PMCID: PMC5936588
PMID: 16075146
DOI: 10.1100/tsw.2005.65
Article ID: 616528
Article version: 1
Subjects: Directions in Science

Modifications of the Glycosaminoglycan-Linkage Region of Proteoglycans: Phosphorylation and Sulfation Determine the Activity of the Human ß1,4-Galactosyltransferase 7 and ß1,3-Glucuronosyltransferase I

Gulberti Sandrine 1
Lattard Virginie 1
Fondeur Magali 1
Jacquinet Jean-Claude 2
Mulliert Guillermo 3
Netter Patrick 1
Magdalou Jacques 1
Ouzzine Mohamed 1
Fournel-Gigleux Sylvie 1 *
1 UMR 7561 CNRS-Université Henri Poincaré Nancy I, Faculté de Médecine, BP 184, 54505 Vandoeuvre-lés-Nancy cedex, France
2 UMR 6005 CNRS, UFR Sciences, Université d'Orléans, 45067 Orléans, France
3 UMR 7036 CNRS-Université Henri Poincaré Nancy I, Faculté des Sciences, 54505 Vandoeuvre-lés-Nancy, France
*Sylvie Fournel-Gigleux: sfg@medecine;uhp-nancy.fr
Publication date: 2005
Electronic publication date: 2005 Jul 12
Volume: 5
First page: 510
Last page: 514
Received 2005 Apr 26; Revised 2005 Jun 15; Accepted 2005 Jun 15
Copyright: Copyright © 2005 Sandrine Gulberti et al.
Copyright year: 2005
License: This is an open access article distributed under the Creative Commons Attribution License, which permits unrestricted use, distribution, and reproduction in any medium, provided the original work is properly cited.
License URL: https://creativecommons.org/licenses/by/3.0/

Keywords: recombinant glycosyltransferases, glycosaminoglycans, phosphorylation/ sulfation, substrate specificity and binding site

Funding: Fonds National pour la Science BMCS152 2004

==============================
Directions in Science
*TheScientificWorldJOURNAL* (2005) 5, 510–514
ISSN 1537-744X; DOI 10.1100/tsw.2005.65

### Modifications of the Glycosaminoglycan-Linkage Region of Proteoglycans: Phosphorylation and Sulfation Determine the Activity of the Human ß1,4-Galactosyltransferase 7 and ß1,3-Glucuronosyltransferase I

Sandrine Gulberti[1], Virginie Lattard[1], Magali Fondeur[1], Jean-Claude Jacquinet[2], Guillermo Mulliert[3], Patrick Netter[1], Jacques Magdalou[1], Mohamed Ouzzine[1], and Sylvie Fournel-Gigleux[1,]*

*[1]UMR 7561 CNRS-Université Henri Poincaré Nancy I, Faculté de Médecine, BP 184, 54505 Vandœuvre-lès-Nancy cedex, France; [2]UMR 6005 CNRS, UFR Sciences, Université d'Orléans, 45067 Orléans, cedex 02, France; [3]UMR 7036 CNRS-Université Henri Poincaré Nancy I, Faculté des Sciences, 54505 Vandœuvre-lès-Nancy, France*

**E-mail**: sandrine.gulberti@medecine.uhp-nancy.fr, vlattard@hotmail.com, magalifondeur@yahoo.fr, jean-claude.jacquinet@univ-orleans.fr, guillermo.mulliert@lcm3b.uhp-nancy.fr, patrick.netter@medecine.uhp-nancy.fr, magdalou@medecine.uhp-nancy.fr,ouzzine@medecine.uhp-nancy.fr, sfg@medecine;uhp-nancy.fr

*Received April 26, 2005; Revised June 15, 2005; Accepted June 15, 2005; Published July 12, 2005*

**KEYWORDS**: recombinant glycosyltransferases, glycosaminoglycans, phosphorylation/ sulfation, substrate specificity and binding site

Proteoglycans (PGs) are macromolecules composed of glycosaminoglycan (GAG) side chains covalently bound to a core protein. There is currently great interest in the elucidation of the biosynthetic pathways of PGs as they are increasingly implicated as important regulators of many fundamental biological processes[1]. These include regulation of cell proliferation and recognition, extracellular matrix deposition, and morphogenesis, which are the consequences of the characteristic GAG moieties interacting with a variety of protein ligands such as growth and/or differentiation factors, cytokines, and morphogens[2].

GAGs include chondroitin/dermatan sulfate (CS/DS) and heparan sulfate/heparin (HS/Hep), which are classified as galactosaminoglycans and glucosaminoglycans, respectively[3]. Major components of these linear GAGs consist of hexosamine (GalNAc, GlcNAc) and hexuronic acid (GlcA or IdoA), which are arranged in alternating sequences to form the so-called repeating disaccharide region. These repeating units contain a number of sulfate substitutents that contribute to the structural and functional diversity of GAGs[4]. Both types of GAGs are covalently bound to serine residues in the core proteins through the common GAG-protein linkage structure GlcAß1,3Galß1,3Galß1,4Xylß1-O-Ser (GlcA-Gal2-Gal1-Xyl-O-Ser)[5,6]. The linkage region tetrasaccharide is formed by the sequential stepwise addition of each sugar residue by respective O-xylosyltransferase I[7], ß1,4-galactosyltransferase 7 (GalT-I)[8], ß1,3-galactosyltransferase 6 (GalT-II)[9], and ß1,3-glucuronosyltransferase I (GlcAT-I)[10]. The transfer of

*Corresponding author.
©2005 with author.

either a αGlcNAc or ßGalNAc residue on the terminal GlcA of the linkage region initiates the polymerization of HS or CS chains, respectively. The synthesis of the common GAG-protein linkage region is a key step in the assembly of PGs because completion of this tetrasaccharide sequence is essential for the conversion of core proteins to functional PGs[5]. In fact, a number of PG precursors are variably (1) substituted with GAG chains, (2) substituted with a tetrasaccharide linkage region, (3) lacking GAG chains, each kind of maturation influencing their biological activity. In addition, PGs such as serglycin, betaglycan, and CD44 can be substituted at a particular attachment site by either a CS or a HS[11]. However, the mechanisms of regulation of these processes are unknown yet.

A number of studies have recently focused on the structure of the linkage region as a possible determinant of GAG chains maturation and sorting[12]. The characterization of the carbohydrate-protein linkage-region fragments from various sources led to the discovery of several structural modifications. The xylose residue can be modified by phosphorylation at C2 position, as shown in the case of aggrecan and decorin. This modification occurs either on HS or DS side chains. On the other hand, the first and the second Gal residues (referred to as Gal1 and Gal2) in the linkage region are sulfated on C4 and/or C6 position and sulfation of the linkage region has only been identified on CS chains. Such modifications have been described for shark and whale cartilage, human urinary trypsin inhibitor, inter-α-trypsin inhibitor, mouse syndecan 1, swarm rat chondrosarcoma, Engelbreth-Holm-swarm mouse tumor, bovine and human articular cartilage ([13,14,15] and for review[16]). In order to shed light on the role of phosphorylation and sulfation in the regulation of the maturation and processing of growing GAG chains, we analyzed in a recent paper the substrate specificity of the glycosyltransferases involved in the synthesis of the linkage region with regard to these modifications. We determined whether the phosphorylation of xylose and sulfation of Gal1 and/or Gal2 affects GalT-I and GlcAT-I activity, respectively. For this purpose, we designed and synthesized C2-phosphorylated xylosides and C4 and/or C6-sulfated digalactose analogs of the GAG-protein linkage region[17]. These compounds have been tested as potential substrates of the two recombinant human enzymes expressed in the yeast *Pichia pastoris*. We found that GalT-I did not exhibit *in vitro* activity towards the C2-phosphorylated xyloside, suggesting that the presence of this modification on the acceptor substrate precludes recognition and/or transfer of Gal onto the xyloside derivative. This result is in agreement with early specificity studies indicating that a xyloside with a bulky 2-O-isopropyl substitutent was not a substrate for native GalT-I from CHO cells[18]. Our observation is consistent with a biosynthetic mechanism in which phosphorylation would occur once Gal is attached to the Xyl residue of the nascent oligosaccharide linkage. On the other hand, as the phosphorylated xylose does not serve as a substrate for GalT-I, it can be suggested that this modification may arrest the biosynthesis of some GAG chains *in vivo* and by a mechanism of phosphorylation/dephosphorylation may regulate the rate of biosynthesis of GAG chains. In fact, inappropriate modifications by sulfation, as shown for thrombomodulin[19], can interfere with the synthesis of the linkage region, producing immature linkage region. However, this issue awaits further investigation.

Furthermore, we examined the influence of sulfation of Gal residues on the activity and specificity of GlcAT-I. This enzyme plays an important role in the completion of the linkage region and has been suggested to be rate limiting in CHO cells[20], skin fibroblasts[21], and chondrocytes[22]. Our recent work suggests that it may represent a pharmacological target in osteoarthritis[22,23]. Interestingly, we demonstrated that the sulfated analog substituted on the C6 position of Gal1 served as a better substrate compared to the unsulfated digalactoside. We found that the enhanced efficiency exhibited by the membrane-bound and by the purified enzyme was due to a higher affinity towards the C6-sulfated digalactoside than its unsulfated counterpart. Site-directed mutagenesis studies allowed us to identify $Trp^{243}$ and $Lys^{317}$ as critical residues of the acceptor substrate binding site. On the other hand, no GlcAT-I activity could be detected towards digalactose when Gal1 was sulfated on the C4 position or when Gal2 was sulfated on the C4 or C6 position. A digalactoside bearing a sulfate substitutent at C6 position of both Gal residues was not substrate of the enzyme either. These results suggest that sulfation is a critical feature to serve or not as a substrate for GlcAT-I. The observation that sulfation influences the activity of the glycosyltransferases involved in GAG synthesis is in agreement with previous observations. Recently,

Seko et al.[24] showed that ß1,4-galactosyltransferase IV involved in the synthesis of keratan-sulfate is specific for GlcNAc-6-O-sulfate. Sato et al.[25] showed strong activity of the recombinant CSGalNAcT-2 towards sulfated chondroitin substrates, suggesting that sulfation stimulates this chondroitin-synthase and possibly elongation of the GAG chains. Furthermore, it is reasonable to hypothesize that similar mechanisms may also regulate the synthesis of HS chains during EXT-catalyzed HS extension, as indicated by the finding that oversulfated HS resulting from glucosaminyl *N*-deacetylase/*N*-sulfotransferase overexpression are longer than wild-type chains[26].

In conclusion, our work demonstrates that phosphorylation and sulfation of the acceptor substrate critically influence the activity of the glycosyltransferases involved in the formation of the common linkage region of PGs, suggesting that these modifications may play a role in the regulation of GAG assembly. The *in vivo* relevance of these findings requires further investigation. In view of the regioselective modifications of the galactose and xylose residues of the tetrasaccharide linkage region by sulfation and phosphorylation and their specific recognition by GlcAT-I and GalT-I, it can be hypothesized that this sequence provides multiple regulatory possibilities driving the biosynthesis of PGs. Careful examination to detect biosynthetic intermediates during the maturation process of a proteoglycan as well as identification of the kinase and the sulfotransferases involved in the modification of the tetrasaccharide linker would represent important steps towards the assessment of the functional significance of phosphorylation and sulfation of the linkage region. Furthermore, our study sheds light on the structure and function of the glycosyltransferases involved in the initial steps of GAG synthesis. This is an important issue with regard to the increasing interest of these enzymes as pharmacological targets. There is growing evidence for the implication of PGs in several disease processes including arthropathies[27], atherosclerosis[28], Alzheimer's disease[29], and cancer[30]. Recently, glycosaminoglycan precursors have been tested with success as antiamyloid[31] antithrombotic[32], and antiproliferative agents[33]. A better understanding of the structure and mechanism of the glycosyltransferases involved in GAG synthesis undertaken in our laboratory[34,35] and in others[7,8,9,10] paves the way towards the development of carbohydrate- and especially GAG-based therapeutics.

### ACKNOWLEDGMENTS

This work was supported by grants from Fonds National pour la Science (ACI "Biologie Cellulaire, Moléculaire et Structurale" BMCS152 2004), IT2B CNRS-INSERM program, Pro-A INSERM program, and PHRC Régional CHU Nancy (France).

This paper was excerpted from the manuscript of Gulberti, S., Lattard, V., Fondeur, M., Jacquinet, J.C., Mulliert, G., Netter, P., Magdalou, J., Ouzzine, M., and Fournel-Gigleux, S. (2005) Phosphorylation and sulfation of oligosaccharide substrates critically influence the activity of human ß1,4-galactosyltransferase 7 (GalT-I) and ß1,3-glucuronosyltransferase I (GlcAT-I) involved in the biosynthesis of the glycosaminoglycan-protein linkage region of proteoglycans. *J. Biol. Chem.* **280**, 1417–1425.

### REFERENCES

1. Linhardt, R.J. and Toida, T. (2004) Role of glycosaminoglycans in cellular communication. *Acc. Chem. Res.* **37**, 431–438.
2. Bernfield, M., Götte, M., Park, P.W., Reizes, O., Fitzgerald, M.L., Lincecum, J., and Zako, M. (1999) Functions of cell surface heparan sulfate proteoglycans. *Annu. Rev. Biochem.* **68,** 729–777.
3. Sasisekharan, R. and Venkataraman, G. (2000) Heparin and heparan sulfate: biosynthesis, structure and function. *Curr. Opin. Chem. Biol.* **4,** 626–631.
4. Kusche-Gullberg, M. and Kjellen, L. (2003) Sulfotransferases in glycosaminoglycan biosynthesis. *Curr. Opin. Struct. Biol.* **13**, 605–611.

5. Sugahara, K. and Kitagawa, H. (2000) Recent advances in the study of the biosynthesis and functions of sulfated glycosaminoglycans. *Curr. Opin. Struct. Biol.* **10,** 518–527.
6. Prydz, K. and Dalen, K.T. (2000) Synthesis and sorting of proteoglycans. *J. Cell Sci.* **113,** 193–205.
7. Götting, C., Kuhn, J., Zahn, R., Brinkmann, T., and Kleesiek, K. (2000) Molecular cloning and expression of human UDP-D-xylose:proteoglycan core protein ß-d-xylosyltransferase and its first isoform XT-II. *J. Mol. Biol.* **304**, 517–528.
8. Almeida, R. Levery, S.B., Mandel, U., Kresse, H., Schwientek, T., Bennett, E.P., and Clausen, H. (1999) Cloning and expression of a proteoglycan UDP-galactose:ß-xylose ß1,4-galactosyltransferase I. A seventh member of the human ß4-galactosyltransferase gene family. *J. Biol. Chem.* **274,** 26165–26171.
9. Bai, X., Zhou, D., Brown, J.R., Crawford, B.E., Hennet, T., and Esko, J.D. (2001) Biosynthesis of the linkage region of glycosaminoglycans: cloning and activity of galactosyltransferase II, the sixth member of the ß1,3-galactosyltransferase family (ß3GalT6). *J. Biol. Chem.* **276**, 48189–48195.
10. Kitagawa, H., Tone, Y., Tamura, J., Neumann, K.W., Ogawa, T., Oka, O., Kawasaki, T., and Sugahara, K. (1998) Molecular cloning of glucuronosyltransferase I involved in the biosynthesis of the glycosaminoglycan-protein linkage region of proteoglycans. *J. Biol. Chem.* **273,** 6615–6618.
11. Esko, J.D. (1991) Genetic analysis of proteoglycan structure, function and metabolism. *Curr. Opin. Cell Biol.* **3**, 805–816.
12. Sugahara, K. and Kitagawa, H. (2002) Heparin and heparan sulfate biosynthesis. *IUBMB Life* **54,** 163–175.
13. Sugahara, K., Ohi, Y., Harada, T., de Waard, P., and Vliegenthart, J.F. (1992) Structural studies on sulfated oligosaccharides derived from the carbohydrate-protein linkage region of chondroitin 6-sulfate proteoglycans of shark cartilage. I. Six compounds containing 0 or 1 sulfate and/or phosphate residues. *J. Biol. Chem.* **267,** 6027–6035.
14. Ueno, M., Yamada, S., Zako, M., Bernfield, M., and Sugahara, K. (2001) Structural characterization of heparan sulfate and chondroitin sulfate of syndecan-1 purified from normal murine mammary gland epithelial cells. Common phosphorylation of xylose and differential sulfation of galactose in the protein linkage region tetrasaccharide sequence. *J. Biol. Chem.* **276,** 29134–29140.
15. Lauder, R.M., Huckerby, T.N., Brown, G.M., Bayliss, M.T., and Nieduszynski, I.A. (2001) Age-related changes in the sulphation of the chondroitin sulphate linkage region from human articular cartilage aggrecan. *Biochem. J.* **358,** 523–528.
16. Krishna, N.R. and Agrawal, P.K. (2000) Molecular structure of the carbohydrate-protein linkage region fragments from connective-tissue proteoglycans. *Adv. Carbohydr. Chem. Biochem.* **56**, 201–234.
17. Jacquinet, J.-C. (2004) An expeditious preparation of various sulfoforms of the disaccharide ß-D-Galp-(1-->3)-D-Galp, a partial structure of the linkage region of proteoglycans, as their 4-methoxyphenyl ß-D-glycosides. *Carbohydr. Res.* **339,** 349–359.
18. Lugemwa, F.N., Sarkar, A.K., and Esko, J.D. (1996) Unusual ß-D-xylosides that prime glycosaminoglycans in animal cells. *J. Biol. Chem.* **271**, 19159–19165.
19. Wakabayashi, H., Natsuka, S., Mega, T., Otsuki, N., Isaji, M., Naotsuka, M., Koyama, S., Kanamori, T., Sakai, K., and Hase, S. (1999) Novel proteoglycan linkage tetrasaccharides of human urinary soluble thrombomodulin, SO4-3GlcAß1-3Galß1-3(+/-Siaa2-6)Galß1-4Xyl. *J. Biol. Chem.* **274**, 5436–5442.
20. Bai, X., Wei, G., Sinha, A., and Esko, J.D. (1999) Chinese hamster ovary cell mutants defective in glycosaminoglycan assembly and glucuronosyltransferase I. *J. Biol. Chem.* **274,** 13017–13024.
21. Takagaki, K., Nakamura, T., Kon, A., Tamura, S., and Endo, M. (1991) Characterization of ß-D-xyloside-induced glycosaminoglycans and oligosaccharides in cultured human skin fibroblasts. *J. Biochem.* **109,** 514–519.
22. Venkatesan, N., Barré, L., Benani, A., Netter, P., Magdalou, J., Fournel-Gigleux, S., and Ouzzine, M. (2004) Stimulation of proteoglycan synthesis by glucuronosyltransferase-I gene delivery: a strategy to promote cartilage repair. *Proc. Natl. Acad. Sci. U. S. A.* **101**, 18087–18092.
23. Gouze, J.N., Bordji, K., Gulberti, S., Terlain, B., Netter, P., Magdalou, J., Fournel-Gigleux, S., and Ouzzine, M. (2001) Interleukin-1ß down-regulates the expression of glucuronosyltransferase I, a key enzyme priming glycosaminoglycan biosynthesis: influence of glucosamine on interleukin-1ß-mediated effects in rat chondrocytes. *Arthritis Rheum.* **44,** 351–360.
24. Seko, A., Dohmae, N., Takio, K., and Yamashita, K. (2003) ß1,4-Galactosyltransferase (beta 4GalT)-IV is specific for GlcNAc 6-O-sulfate. ß4GalT-IV acts on keratan sulfate-related glycans and a precursor glycan of 6-sulfosialyl-Lewis X. *J. Biol. Chem.* **278,** 9150–9158.
25. Sato, T., Gotoh, M., Kiyohara, K., Akashima, T., Iwasaki, H., Kameyama, A., Mochizuki, H., Yada, T., Inaba, N., Togayachi, A., Kudo, T., Asada, M., Watanabe, H., Imamura, T., Kimata, K., and Narimatsu, H. (2003) Differential roles of two N-acetylgalactosaminyltransferases, CSGalNAcT-1, and a novel enzyme, CSGalNAcT-2. Initiation and elongation in synthesis of chondroitin sulfate. *J. Biol. Chem.* **278,** 3063–3071.
26. Pikas, D.S., Eriksson, I., and Kjellen, L. (2000) Overexpression of different isoforms of glucosaminyl N-deacetylase/N-sulfotransferase results in distinct heparan sulfate N-sulfation patterns. *Biochemistry* **39**, 4552–4558.
27. Schwartz, N.B. and Domowicz, M. (2002) Chondrodysplasias due to proteoglycan defects. *Glycobiology* **12,** 57–68.
28. Shriver, Z., Liu, D., and Sasisekharan, R. (2002) Emerging views of heparan sulfate glycosaminoglycan structure/activity relationships modulating dynamic biological functions. *Trends Cardiovasc. Med.* **12**, 71–77.

29. van Horssen, J., Wesseling, P., van den Heuvel, L.P., de Waal, R.M., and Verbeek, M.M. (2003) Heparan sulphate proteoglycans in Alzheimer's disease and amyloid-related disorders. *Lancet Neurol.* **2**, 482–492.
30. Wegrowski, Y. and Maquart, F.X. (2004) Involvement of stromal proteoglycans in tumour progression. *Crit. Rev. Oncol. Hematol.* **49**, 259–268.
31. Kisilevsky, R., Szarek, W.A., Ancsin, J., Vohra, R., Li, Z., and Marone, S. (2004) Novel glycosaminoglycan precursors as antiamyloid agents. IV. *J. Mol. Neurosci.* **24**, 167–172.
32. Martin, N.B., Masson, P., Sepulchre, C., Theveniaux, J., Millet, J., and Bellamy, F. (1996) Pharmacologic and biochemical profiles of new venous antithrombotic ß-D-xyloside derivatives: potential antiathero/thrombotic drugs. *Semin. Thromb. Hemost.* **22,** 247–254.
33. Mani, K., Havsmark, B., Persson, S., Kaneda, Y., Yamamoto, H., Sakurai, K., Ashikari, S., Habuchi, H., Suzuki, S., Kimata, K., Malmstrom, A., Westergren-Thorsson, G., and Fransson, L.A. (1998) Heparan/chondroitin/dermatan sulfate primer 2-(6-hydroxynaphthyl)-O-ß-D-xylopyranoside preferentially inhibits growth of transformed cells. *Cancer Res.* **58**, 1099–1104.
34. Ouzzine, M., Gulberti, S., Netter, P., Magdalou, J., and Fournel-Gigleux, S. (2000) Structure/function of the human Ga1ß1,3-glucuronosyltransferase. Dimerization and functional activity are mediated by two crucial cysteine residues. *J. Biol. Chem.* **275**, 28254–28260.
35. Ouzzine, M., Gulberti, S., Netter, P., Magdalou, J., and Fournel-Gigleux, S. (2002) The donor substrate specificity of the human ß1,3-glucuronosyltransferase I (GlcAT-I) towards UDP-glucuronic acid is determined by two crucial histidine and arginine residues. *J. Biol. Chem.* **28,** 25439–25445.

**This article should be referenced as follows:**

Gulberti, S., Lattard, V., Fondeur, M., Jacquinet, J.C., Mulliert, G., Netter, P., Magdalou, J., Ouzzine, M., and Fournel-Gigleux, S. (2005) Modifications of the glycosaminoglycan-linkage region of proteoglycans: phosphorylation and sulfation determine the activity of the human ß1,4-galactosyltransferase 7 and ß1,3-glucuronosyltransferase I. *TheScientificWorldJOURNAL* **5**, 510-514.

**Handling Editor:**

M. Gotte, Principal Editor for *Cell Biology* and Editorial Board Member for *Biochemistry* and *Molecular Biology* — domains of *TheScientificWorldJOURNAL*.

### BIOSKETCH

**Sylvie Fournel-Gigleux** is Director of Research at INSERM (Institut National de la Santé et de la Recherche Médicale, France). Her research group in the UMR 7561-CNRS University of Nancy 1 is dedicated to the investigation of the structure-function relationships of UDP-glycosyltransferases. She is involved in the study of the UDP-glucuronosyltransferases responsible for drug metabolism and cell homeostasis. She also focuses on the structural and functional aspects of the glycosyltransferases responsible for the biosynthesis of glycosaminoglycans and their implications in pathological processes such as osteoarthritis.
